# The Looming Threat: Cancer in Sub‐Saharan Africa

**DOI:** 10.1002/onco.13963

**Published:** 2021-09-29

**Authors:** Alfred I. Neugut, Wafaa M. El‐Sadr, Paul Ruff

**Affiliations:** ^1^ Department of Medicine and Herbert Irving Comprehensive Cancer Center, Vagelos College of Physicians and Surgeons, and Department of Epidemiology, Mailman School of Public Health, Columbia University New York New York USA; ^2^ ICAP at Columbia University New York New York USA; ^3^ Division of Medical Oncology, University of Witwatersrand Faculty of Health Sciences Johannesburg South Africa

## Abstract

Recent trends in cancer epidemiology in low‐ and middle‐income countries show the need for urgent action. This article focuses on sub‐Saharan Africa, where populations are showing an increased risk for diseases associated with the Western lifestyle, including cancer.

Concerns regarding the health and well‐being of populations of low‐ and middle‐income countries (LMICs), particularly those of sub‐Saharan Africa (SSA), have typically focused on problems that many in the U.S. and other high‐income countries (HICs) consider diseases of the past. For many countries in sub‐Saharan Africa, infectious diseases like HIV, tuberculosis, and malaria, along with the consequences of malnutrition and challenges in maternal and child health, whereas for HICs, chronic noncommunicable diseases of middle and older ages—cancer, coronary artery and cerebrovascular disease, diabetes, and dementia—pose major threats to the health of their populations. However, in SSA, the picture is slowly but surely evolving, with the threat of noncommunicable diseases, including cancers, now looming large [1].

This transformation is attributable to two parallel phenomena observed in that part of the world. The first is the impact of the scale‐up of effective antiretroviral therapy for HIV, resulting in a truly remarkable transformation of HIV from “a death sentence” to a chronic, manageable condition, as well as substantial progress in malaria control and in decreases in maternal and child mortality. Advances in the management of HIV have resulted in a substantial increase in life expectancy among people living with HIV in SSA, with such individuals now surviving into middle and older ages. The aging of this population is a critical factor in their increased susceptibility to noncommunicable diseases [2, 3].

At the same time, many LMICs, including those in SSA, are experiencing economic growth. Although profound disparities in wealth remain, this economic growth, which is welcomed, has resulted in fundamental changes in lifestyles, with increase in caloric intake, tobacco smoking, and sedentary status. These realities put sub‐Saharan African populations at increased risk for diseases that are associated with the Western lifestyle, notably certain cancers, coronary heart disease, stroke, and diabetes. Tobacco use has been on the rise, partially caused by tobacco companies targeting LMICs because of loss of sales in HICs, as has obesity, due to increased caloric intake combined with a more sedentary lifestyle. As urbanization has accelerated, many have left behind more active lifestyles in rural areas, and have adopted more meat rich “fatty” diets associated with depletion of fresh vegetable/fruit “high fiber” diets [4]. All these lifestyle and health access changes have contributed toward an increased risk of chronic noncommunicable diseases [5].

Mayosi and colleagues have reported that in South Africa, home to the largest population of people living with HIV in the world (approximately 23%), the recent decades have seen a rise in chronic noncommunicable disease rates attributable to the changes in risk factors noted above. In particular, they reported that between 1999 and 2006, the mortality rate from prostate cancer increased by 12%, whereas breast cancer mortality increased by 21% [5]. More recent studies have suggested that these increases in cancer rates have continued and have been observed for other malignancies as well [6]. Increases have also been noted throughout SSA for incidence for various major cancers, including breast, colorectal, lung, and prostate cancers. Recent increases in colorectal cancer incidence rates in South Africa, especially in younger patients, have paralleled increases in socioeconomic changes among ethnic groups [7]. Others have recently suggested that as countries develop economically, the types of cancers observed also evolve from those that are infection‐related to those that are not infection‐related [1].

These recent trends in cancer epidemiology compel the need for urgent action. There is an urgent need to put measures in place to address the risk factors for various cancers in order to stem the rise in cancer rates. Unfortunately, tobacco use is increasing in SSA as it stands more than 30% in that region as compared with <15% in the U.S., where lung cancer incidence and mortality rates are falling dramatically. A serious effort to stem the use of tobacco, particularly given the reported increased susceptibility of persons living with HIV to its carcinogenic effects, is called for [8]. The high rates of cervical cancer and other squamous cell malignancies must motivate the urgent necessity to expand vaccination programs for human papillomavirus as well as for hepatitis B virus for the prevention of hepatocellular carcinoma in endemic areas. Vigorous efforts are needed to expand community informational campaigns and provider education regarding cancer prevention measures, such as tobacco cessation, calorie control, and obesity prevention. Although routine cancer screening has proved to be an important cancer control modality for certain cancers in HICs, thus far, the one cancer for which there has been screening efforts in SSA is cervical cancer. With almost no breast or colon cancer screening being conducted in the region, this has limited the information on rates of these cancers. The higher rates of breast cancer in high‐income countries may reflect, in part, the increased use of breast cancer screening programs [9]. The costs of mammography or colorectal screening are substantial and beyond the reach of most individuals and programs in SSA. Their use for high‐risk groups may need to be prioritized in this context.

Of equal importance is the need for enhanced capabilities for diagnosis and management of cancers in the region. In many ways, although HIV is now considered a chronic manageable condition, cancer is often thought of as a “death sentence.” As things stand now, there are limited infrastructure and capabilities for cancer diagnosis and care, such as limited radiation therapy accelerators, inadequate pathology services, palliative and hospice, and social support systems, a paucity of well‐trained oncologists and oncology nurses, and a dearth of the resources to secure access to an ongoing supply of modern cost‐effective cancer chemotherapeutic agents.

Faced with this threat, combined with limited prioritization of cancer as a public health challenge in SSA and the lack of resources and programs to confront it, we urge that a comprehensive health systems focused approach be adopted to address these gaps. Table 1 shows examples of priority recommendations by health system building blocks, that is, financing, governance, laboratory, commodities, human resources, information systems, service delivery and community.

**Table 1:** Illustrative supportive interventions by health system building block

Health System Building Blocks	Possible interventions
Financing	Investment from bilateral and multilateral sources
	Prioritization of cancer programming in national health funding decisions
	Subsidizing of cost of cancer treatments
Governance and policy	Establishment of Cancer Task Force at Ministries of Health
	Development of national cancer strategic plans
	Engagement of community representatives in shaping cancer policy and programs
	Support for research initiatives to address knowledge gaps
Information systems	Development of national cancer registries/surveillance systems in line with the International Agency for Research on Cancer
	Establishment of case‐based management systems for cancer cases
Laboratory	Increased availability of pathology diagnostic services
	Establishment of virtual pathology consultation networks
Commodities	Increased availability of newer chemotherapeutic drugs and analgesics for pain relief in line with the latest WHO Essential Medicines List
Service delivery	Development of regional networks and agreements between countries to establish pathways of care
	Establishment of comprehensive cancer centers of excellence at national and provincial levels
	Establishment of adequate radiotherapy and chemotherapy services
	Support for comprehensive case management models of care
Human resources	Training and mentorship of health care workers (e.g., physicians, nurses, pharmacists) in cancer prevention, diagnosis, and care
	Use of virtual learning platforms
	Development of a cadre of peer support workers for patients with cancer
	Linkage of facility and community care systems
Community	Development of cancer sensitization and prevention campaigns
	Support for community‐based organization that support cancer patients and their families

Abbreviation: WHO, World Health Organization.

The global COVID‐19 pandemic offers some important lessons learned, particularly with regard to virtual learning for health providers, as well as opportunities for the dissemination of information and provision of mentorship and supportive supervision [10, 11].

The global mobilization that focused on addressing HIV, tuberculosis, and malaria has made an enormous difference in the lives of people in SSA. However, SSA now faces a double health threat, the continued threat of conditions that have prevailed for decades in LMICs and are now facing those that dominate in HICs. Cancer is emblematic of these new threats. However, there is reason for optimism, as countries in SSA are poised to adapt the strategies they developed to address communicable disease can be adapted to respond to cancer and other noncommunicable diseases. But the reality is that it will take a strong will, national leadership, resource mobilization, and a commitment to working together across continents and borders to advance the health and well‐being of the people living south of the Sahara Desert as they face their next health challenge.

## Disclosures

**Alfred I. Neugut:** Otsuka, GlaxoSmithKline, Eisai, United Biosource Corporation (C/A), Otsuka (RF), Hospira (ET), EHE, Intl (SAB). The other authors indicated no financial relationships.

(C/A) Consulting/advisory relationship; (RF) Research funding; (E) Employment; (ET) Expert testimony; (H) Honoraria received; (OI) Ownership interests; (IP) Intellectual property rights/inventor/patent holder; (SAB) Scientific advisory board
